# In-vitro mapping of E-fields induced near pacemaker leads by simulated MR gradient fields

**DOI:** 10.1186/1475-925X-8-39

**Published:** 2009-12-15

**Authors:** Howard I Bassen, Gonzalo G Mendoza

**Affiliations:** 1 Division of Physics, Office of Science and Engineering Laboratories, Center for Devices and Radiological Health, FDA, 10903 New Hampshire Avenue Silver Spring, MD 20993-0002, USA; 2 School of Engineering, The Catholic University of America, Washington, DC 20064, USA

## Abstract

**Background:**

Magnetic resonance imaging (MRI) of patients with implanted cardiac pacemakers is generally contraindicated but some clinicians condone scanning certain patients. We assessed the risk of inducing unintended cardiac stimulation by measuring electric fields (E) induced near lead tips by a simulated MRI gradient system. The objectives of this study are to map magnetically induced E near distal tips of leads in a saline tank to determine the spatial distribution and magnitude of E and compare them with E induced by a pacemaker pulse generator (PG).

**Methods:**

We mapped magnetically induced E with 0.1 mm resolution as close as 1 mm from lead tips. We used probes with two straight electrodes (e.g. wire diameter of 0.2 mm separated by 0.9 mm). We generated magnetic flux density (B) with a Helmholtz coil throughout 0.6% saline in a 24 cm diameter tank with (dB/dt) of 1 T/sec (1 kHz sinusoidal waveform). Separately, we measured E near the tip of leads when connected to a PG set to a unipolar mode. Measurements were non-invasive (not altering the leads or PG under study).

**Results:**

When scaled to 30 T/s (a clinically relevant value), magnetically-induced E exceeded the E produced by a PG. The magnetically-induced E only occurred when B was coincident with or within 15 msec of implantable pacemaker's pulse.

**Conclusions:**

Potentially hazardous situations are possible during an MR scan due to gradient fields. Unintended stimulation can be induced via abandoned leads and leads connected to a pulse generator with loss of hermetic seal at the connector. Also, pacemaker-dependent patients can receive drastically altered pacing pulses.

## Background

Magnetic Resonance Imaging (MRI) systems expose patients and nearby clinical personnel to intense, low-frequency, pulsed magnetic fields [1] as well as static magnetic fields and RF fields. The magnetic fields of interest in this study are produced by the gradient coils and associated electronics. Most MRI systems produce magnetic fields from three separate gradient fields with a maximum rate of change (slew rate) of 30 T/s to 180 T/s. The fields from each gradient coil are uniform in one plane (such as a transverse slice of the body). These-fields induce electric fields in conductive media such as patients' bodies in the bore of the magnetic resonance (MR) system or in the bodies of persons close to the MR system. The induced fields can cause nerve stimulation. If conductive wires are in patients or nearby persons, the induced E-fields near the ends of the wires will be many times greater than if the wires were not present. For this reason among others, patients are generally not allowed (by present practices) to undergo MRI procedures if they have implanted cardiac and neurological stimulations devices, or other medical devices that consist, in part, of electrically-conductive objects in their body. However, there is at present a trend to produce medical implants that can be MRI compatible so that patients with these implants could be imaged. In addition, some advocate the scanning of patient with conventional medical implants such as cardiac pacemakers, using special protocols [2-4].

**Figure 1:**
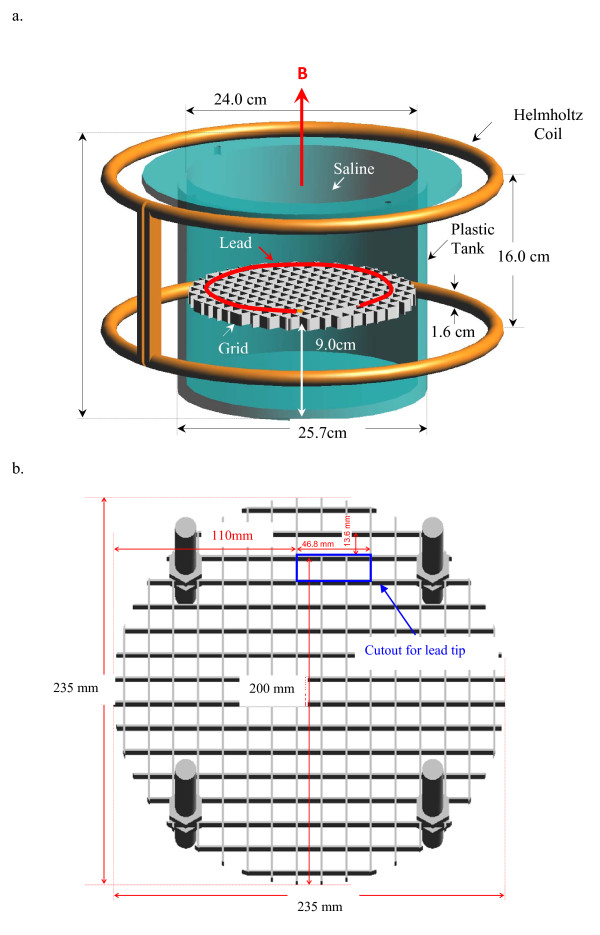
**a. Saline tank and Helmholtz coil**. **b**. Plastic support grid

It is well known that when conductive (metal) objects in the body or saline are exposed to time varying magnetic fields the induced electric (E) fields are highest near sharp edges and at the ends of conductive objects such as wires. Results of many studies have been published on heating at the ends of implanted wires and leads due to E-fields induced by the RF coil of MRI systems [5-7]. This heating is highly localized, i.e., focused within a few cubic millimeters and is therefore difficult to measure accurately [7]. However, there is little information published on the magnitude and spatial distribution of the E-field surrounding the tips of electrodes of medical implants such as cardiac and neural stimulation leads in bodies of patients exposed to MRI gradient fields. We performed measurements in-vitro in a 24 cm diameter cylindrical tank filled with saline (Fig. 1a) that served as a simple model of the human torso. We exposed the saline tank and various simulated and real cardiac pacemaker leads to a simulated MRI gradient field [8]. The purpose was to determine the magnitude and spatial distribution of the E-field induced in the saline immediately adjacent to the objects during exposures to simulated MRI gradient fields. Compared to clinical exposures of patients, our induced fields were higher for a given rate of change of B field. Our exposure conditions were to a spatially uniform B field and our patient model had a single value of conductivity throughout. The results of these measurements enabled us to estimate if the induced E-field at the tips of pacemaker leads exceeded the therapeutic levels that are delivered to patients by conventional cardiac pacemakers.

## Background theory of magnetically induced electric fields in electrically conductive media

An E-field is induced in a volume of conductive medium (such as a tank filed with saline solution or the human body) whenever a time varying magnetic field passes through the medium. The E-field is a vector quantity with magnitude and direction. We used an exposure geometry with a cylindrical container (Fig. 1) where the B field (magnetic flux density) is perpendicular to the surface of the saline in a tank. The saline conductivity was 1.0 S/m. The resulting induced E-fields in the saline are in the form of concentric circular rings, in a plane that is parallel to the surface of the saline. The magnitude of the E-field increases as the radial distance from the center increases. The direction of the E-field vector is normal to a radial line drawn for the center of the tank to the outer circumference. The value of the E-field in the center of the tank is zero. Equation 1 defines the root mean square (RMS) magnitude of the E-field at any point in the saline induced by a sinusoidally varying B field. This simple equation is derived from Faraday's law and was verified using dipole measurement system in a saline tank [9]. In this paper, the E-field is an RMS value for all magnetically induced fields.

Where:

*E *= electric field at a point (V/m) in the saline

*f *= frequency (Hz) of the magnetic field

*r *= radial distance from center of saline (meter)

*B *= Magnetic flux density (Tesla)

When a conducting wire with or without insulation is placed into a saline tank and exposed to a magnetic field, a current is induced in the wire. If both ends of an insulated wire have a small region of insulation removed this will produce an enhanced E-field in the saline. The E-field is greatest very close to the tip of the wire. The current density (J) at any point the surrounding saline is defined in equation 2.

Where:

*J *= current density (amps/meter^2^)

*σ *= electrical conductivity of the saline (S/m)

*E *= RMS E-field at the point of interest (V/m)

If the proximal end is not in good electrical contact with the saline, current will not be induced in the lead and very little E-field will exist at the distal tip.

A time varying B field will induce a voltage (V_b_) between the two ends of a wire that is configured so it forms a loop that is not completely closed (short circuit). Equation 3 defines the V_b _induced across a gap between the ends of a single turn loop of insulated wire, with only its ends not insulated. The voltage is equal to the line integral of the E-field over the path. This voltage is in turn equal to the integral of the component of the rate of change of the magnetic flux density that is normal to and passes through the planar surface formed by the loop with an area A.

Where:

*E *= electric field (V/m)

*dl *= unit element of a closed loop

*dB/dt *= rate of change of the magnetic flux density (Tesla/s)

*dA *= unit element of the area A

**Figure 2:**
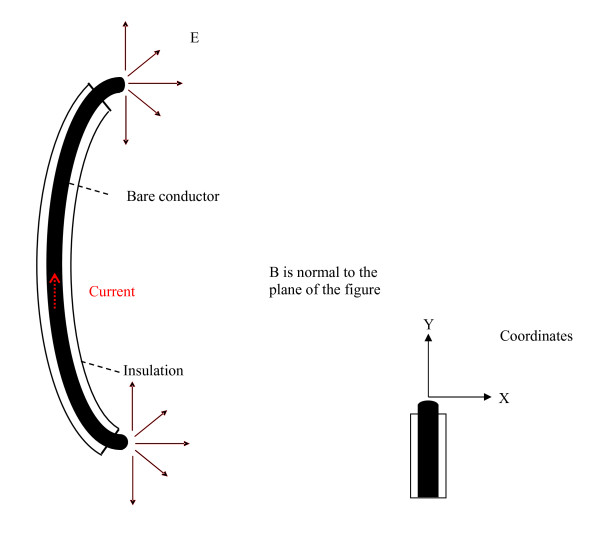
**Cross section of the center of an insulated conductor with bare ends**. The E- vector points in a direction normal to the surface of the conductor

**Figure 3:**
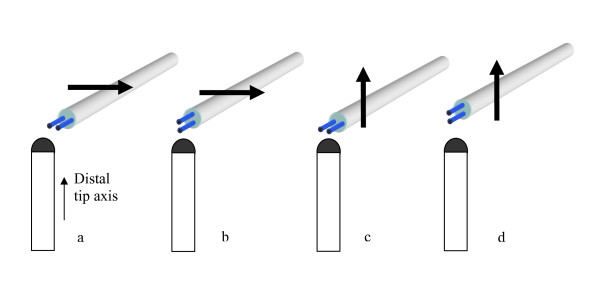
**Probe-tip orientation terminology is defined as follows**. For each of these scans (perpendicular and parallel) we took data for two probe-tip orientations. **a.** scan perpendicular, probe tips normal to distal tip axis **b**. scan perpendicular, probe tips aligned to distal tip axis **c.** scan parallel, probe tips normal to distal tip axis **d.**  scan parallel , probe tips aligned to distal tip axis.

For the case of a thin, long conductive object submerged in saline, the direction of the E vector induced by magnetic induction or by injected voltage is as follows. This assumes the object is much more conductive than the surrounding saline. The E-field vector points in a direction normal to the surface of the conductor (Fig. 2). The conductor is insulated except for the two "bare" ends. The figure is drawn in the x-y plane containing the 'center cut' of the conductor. If the E-field is measured with a two-electrode probe, then its magnitude and direction must be determined by measuring each of the three components of the field. This is done by taking three separate measurements with the probe electrodes oriented in perpendicular directions at the same point. Figure 3 shows two of the three possible orthogonal orientations of the probe. The third orientation is perpendicular to these two orientations in the Z direction. Once the separate components of the E-field are measured, the vector sum can be calculated to get the true magnitude of the field as shown in equation 4. For our symmetric system, the Z component of the E-field is zero in the x-y plane containing the center cut of the conductor. Therefore, we did not measure this component. The Y component is zero anywhere along the central axis of the conductor (X = 0, Z = 0) as long as the conductor is centered in the X-Y plane.

Where:

 = magnitude of the E vector and the x, y and z components of the vector E are listed in the equation.

## Methods

### E-field probe

**Figure 4:**
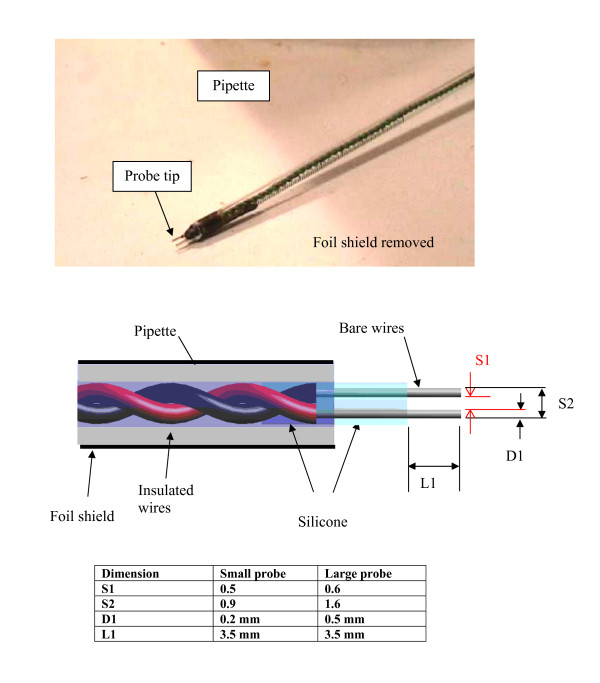
**The 2-electrode probe**.

We measured E-fields with two versions of a two-electrode probe made with solid conductor copper wires. The large probe used American Wire Gauge (AWG) 24 with a diameter of 0.5 mm. The small probe used AWG 32 wire with a diameter of 0.2 mm. The tips of the wires of the large probe were separated by 1.6 mm for the outside dimension and 0.6 mm for the inside dimension (Fig. 4). For the small probe the outside and inside separations were 0.9 and 0.5 mm respectively. The wires were placed in a disposable plastic, standard-tip, serological 10 mL graduated pipette (length: 22.5 cm, inner diameter: 2.5 mm, outer diameter: 4.5 mm). At the distal tip of the probe the bare wires of each probe extended 3.5 mm from the tube and were held in the tube with aquarium grade silicone gel. Each probe had a twisted pair of insulated wires inside the tube (the same wire that the probe was constructed with), that extended 6 meters. These wires were connected at the proximal end to a preamplifier of our data acquisition system. The wires going from the glass tube to the preamplifier were oriented in a straight line parallel to the B field vector of the gradient coil. For certain experiments we needed to provide a reduction in the pickup of electric fields in the measurement environment. For these measurements we added an aluminum foil shield around the glass tube and connected it to our measurement system "ground".

### Gradient field simulator

To generate a 1 kHz B field with 1.25 Gauss RMS (1.25 × 10^-4 ^T) we used a modified Helmholtz coil with an outer diameter of 41.6 cm. This device has two single-turn coils each made of copper tubing. The tubing diameter is 1.6 cm. The broad surfaces of the two coils were in parallel planes separated by 14.6 cm (Fig. 1). The coils were connected in parallel. This device was originally designed for producing a spatially uniform field to test implanted medical devices in saline for susceptibility to electromagnetic interference [10]. The coil was driven by current from a transconductance amplifier (Clarke-Hess, Medford, NY 11763, USA). The input to the amplifier was a 1 kHz sinusoidal voltage from a Hewlett Packard Arbitrary Waveform Generator (Model 33120A, Palo Alto CA, USA). The B field throughout the volume between the coils was measured with a magnetic field probe with dimensions of 3.7 × 3.7 × 10 cm (ERM model 1850, ERM Pittsburgh, PA, USA). This field is linearly polarized and is intended to provide a simplified but worst-case replica of one axis of the gradient field used for a clinical MRI system with a smaller diameter than a clinical MRI coil. Our field reverses polarity every 1 ms, creating a rate of change of B that closely approximates a 1 T/s ramp at portions of the sinusoid that are within ± 45 degrees of the zero crossings of the waveform.

Our coil produced a spatially uniform B field (Z-oriented) throughout a cylindrical volume. This allowed us to produce an induced E-field in the saline tank and surrounding the tip of a pacemaker lead that was well characterized. Results of measurements made using this simulator can be extrapolated to the E-field produced a gradient coil of an MRI system. The sinusoidal field we used differs from the gradient field of a clinical MRI in that it is a continuous wave and not a series of pulses. However, the use of a sinusoid has the distinct advantage that the induced E and J in saline tank can be calculated simply and exactly. Another difference is that a clinical MRI system has three gradient coils, each producing a magnetic field in one of three orthogonal directions. These fields are intentionally non-uniform in terms of the spatial distribution of the B field over the volume patient's body. However, most gradient coils produce a uniform B field in a single plane in the body, for example the plane containing the entire lead of an implanted pacemaker. Our coil produces this condition and produces a worst-case situation by producing a uniform field over the entire volume, not just in a single plane in the body. Therefore, the entire pacemaker lead is exposed to the maximum B field, which may not be the case in a clinical situation.

### Patient simulating tank

We used a cylindrical plastic tank, filled with saline to evaluate the E-field induced in a patient's body (Fig. 1). The tank had an inner diameter of 24 cm and a height of 22.7 cm and was centered inside the Helmholtz coil. The tank was filled with saline (0.6% NaCl) to a depth of 15 cm. This produced a mixture with salinity in the physiological range and having conductivity with a simple, convenient value of 1.0 S/m. The tank was constructed of clear acrylic plastic cylinder 24.1 cm (9.5 in) outer diameter, 0.6 cm wall thickness (0.25 in), depth 24.1 cm (9.5 in). A plastic support grid was mounted horizontally above the bottom of the saline tank. The plastic grid was cut from a fluorescent light fixture cover made of nonconductive plastic which is cut to fit the box's opening so that the grid's top surface is placed 9 cm above the bottom of the tank on four plastic threaded bolts screwed into plastic bolts on the grid. The grid is constructed of beams 0.2 cm wide, 0.9 cm thick and spaced 1.4 cm apart. The grid walls are 1.3 cm on a side. This grid was used for testing pacemakers for electromagnetic interference in ANSI/AAMI standard PC69 [11]. The leads and pulse generator of pacemakers or implantable defibrillators rest on this grid. A cutout on one location at the outer edge of the grid (46.8 × 13.6 mm) removed all plastic from the immediate proximity of the distal tip of a lead under test (Fig. 1b).

### Probe scanner

Spatial mapping of E-fields was performed by moving the probe in a straight line through areas of interest. We scanned areas of interest in the saline tank using a SPEAG DASY5 robotic Dosimetric Assessment System (Schmid & Partner Engineering AG, Zeughausstrasse 43, CH - 8004 Zurich Switzerland) which is referred to in this paper as the 'scanner'. This device has a 6-axis industrial robotic arm with a 0.1 mm linear motion step size in three dimensions and the capability to move and record tip location anywhere within more than 100 cm × 50 cm horizontal plane and vertically more than 100 cm. We scanned continuously at a constant speed in a single direction. For a scan over a 10 mm linear path near a lead tip we set the scanner to pause and take probe voltage readings every 0.1 mm. For scans across the entire diameter of the tank we set the scanner to take readings every 2 mm. During each pause, 15,000 voltage readings were taken and the RMS value was computed over many cycles of the B field. The probe was placed so the pipette holding the two wires of the distal end was oriented in a vertical direction and the twisted pair of wires was also vertical (parallel to the B vector for minimum coupling). Linear scans were performed in selected locations in a horizontal plane to measure the E-field in the saline near the pacemaker leads as well as in the saline with no leads present.

### Data acquisition instrumentation

Our data acquisition system consisted of the following hardware components. The probe output wires were fed to a Grass model P55 AC Preamplifier (Grass technologies, West Warwick, RI 02893 U.S.A.). The amplifier was used with a 300 - 3000 Hz bandpass setting and a gain of 1000. For certain measurements, when a pacemaker pulse generator was connected to the leads under test, a DC to 3000 Hz passband was used. The signal out of the preamplifier was delivered to the SPEAG system signal input consisting of a National Instruments PXI 6115 data acquisition system with 4 analog input channels each with a 12-bit analog to digital converter operating at 15,000 samples/second. The 1 kHz sinusoidal voltage output from the probe and its pre-amplifier were converted to an RMS value for each measurement point (nominally 0.1 mm). For measurements of pacemaker pulse generator waveforms we used a digital oscilloscope (Lecroy, model LS140, 700 Chestnut Ridge Road NY 10977-6499, USA) instead of the SPEAG data acquisition system.

It should be noted that our probe and measurement system may not be able to measure or estimate the magnitude of certain clinical gradient-field induced E-fields near lead tips. Specifically pulses from clinical MRI systems with very fast rise times will be attenuated by the bandpass filters we used to reduce measurement noise.

### Calibration of the E-field measurement probes

Calibration of the two-electrode probe was performed by measuring the voltage across the two electrodes of our probe (at the proximal end of the wires) when the distal tip was placed in a region in the saline tank with a known value of the E-field. We exposed the saline tank to a known value of a 1 kHz B field (1.25 × 10^-4 ^T) and then calculated the induced E-field in a region where the spatial distribution of the E-field was virtually constant, relative to the probe electrode dimensions. The two probe electrodes were oriented for maximum coupling with the E-field. The calculated E-field was 0.047 V/m at the outer edge of the saline (radius = 12 cm). The two-electrode probe was then assigned a calibration factor based on the measured voltage between the electrodes.

Another test was made to assess the quality of our E-probe measurement system (including signal wires and preamplifiers). We measured the unwanted electromagnetic pickup of voltages in the twisted pair of wires between the probe's distal tip at one end and the preamplifier in the measurement system at the other end. We determined this unwanted pickup or interference by measuring the E-field at the exact center of the saline tank. At this location the E-field is zero (this corresponds to radial distance r = 0 in Eqn. 1). In fact the measured value of the E-field with the two-electrode probe was not distinguishable from the noise level of our measurement system. This noise level was less than 2% of the voltage measured at the outer edge of the saline-filled tank. Therefore, we had a minimal measurement artifact due to pickup.

### Pacemaker leads

We used two pacemaker leads and a "simulated lead" in our study. The leads were individually placed in a circular arrangement on the support grid in the saline-filed tank. For the simple, "simulated lead" we used a solid conductor copper wire, 0.6 mm diameter wire, (AWG 22), with 0.4 mm thick insulation, with a 54 cm length. The insulation was removed for the last 5 mm of the proximal end. The distal end was completely covered with insulation, but this end was bluntly cut so that the circular cross section of the end of the metallic wire was exposed to saline. We found that a bluntly cut wire (rather than a wire with an exposed conductor with length greater than 0.5 mm or more) produced an E-field near the distal tip that was much higher and was most similar to the E-field near the tip of a real pacemaker lead.

**Figure 5:**
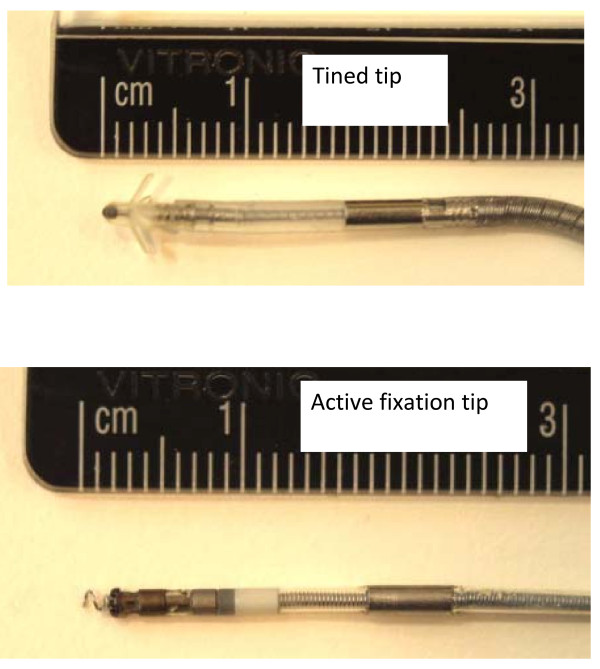
**Pacemaker lead tips**.

We measured the E-field induced at the distal tip of this simulated lead plus the distal tips of two commercially produced pacemaker leads. One lead had a tined tip. This was a Medtronic model number 5554 Capsure Z high impedance atrial J lead with silicone insulation (Medtronic, 710 Medtronic Parkway, Minneapolis, MN 55432-5604 USA). The lead was 54 cm long and had an outer diameter of 1.6 mm. This lead was insulated except for the electrode at the distal tip and a bipolar ring electrode. The tip had an exposed electrically conductive hemisphere with a diameter of approximately 0.5 mm. The proximal end had a solid metal connector in the shape of a cylindrical rod that was used to insert and connect the lead to a pacemaker pulse generator. This connector had a diameter of 1.6 mm and a length of 5.2 mm. This lead had a second bipolar ring electrode near the distal end that was not evaluated in terms of magnetically induced E-fields. We also measured the E-field at the end of a commercially produced pacemaker lead with an active fixation screw tip. This was a Guidant Fineline II lead, with polyurethane insulation (Boston Scientific Corporate Headquarters, One Boston Scientific Place, Natick, MA 01760-1537 USA). This lead was 58.4 cm long and 1.5 mm diameter. The active fixation tip was an extremely small helical coil with a wire diameter of approximately 0.2 mm. The proximal end had a solid metal connector (in the shape of a cylindrical rod). The connector had a diameter of 1.6 mm and a length of 5.2 mm. For each of the commercial pacemaker leads the metal connector at the proximal tip was exposed to saline, creating a closed electrical path (loop) so magnetically induced currents could flow through both ends of the lead and the saline. The tips of each commercially produced pacemaker lead are shown in figure 5.

### Lead configurations

We configured each of three leads (simulated, tined tip and active fixation tip) in a circular loop with its insulated conductor routed along the outer circumference of the plastic grid in the saline tank with the same loop radius. The simulated lead was configured in a circular path with a diameter of 19.75 cm. The gap between the distal and proximal ends for this loop was 5 cm. The area enclosed by this loop is on the order of 300 square cm. This is similar to the worst case loop area of 314 cm^2 ^identified in the AAMI pacemaker EMC standard [11].

### Pacemaker pulse generator

For some experiments we used one of two cardiac pacemaker pulse generators. One was a Medtronic KDR401 set to unipolar pacing with a "typical" 0.5 V pulse. We also used a Guidant "Insignia" pacemaker set to a "minimum" pacing level of 0.1 V. The waveforms of the pulses are illustrated in the results section of this paper. The generator was connected to the tined tip lead. This enabled us to compare the absolute value of "injected" E-field in the saline at the distal tip to the magnetically induced E-field at this same location. The injected E-field is produced by the pulse generator, in the lead via conduction. This 'injected" E-field represents what is produced in a patient near the tip of when the gradient field is off, i.e., it is the normal therapeutic stimulation. In contrast, the "magnetically induced" E-field is produced by the gradient field.

### Mapping magnetically induced E-fields

We used the scanning system and E probes to measure the E-field along a series of linear paths that passed directly in front of the distal tips of various pacemaker leads. We used both the large and small probes to determine the effects of probe tip size on sensitivity, measured spatial resolution and field patterns. This location is where the E-field is highest. We defined special terminology to describe the *path *we used to scan the probe past a pacemaker lead under test and to define the *orientation *of the probe electrodes with respect to the pacemaker lead under test (Fig. 3). We also defined a *"distal tip axis" *and a *"probe-tip orientation." *Each lead was configured to lie in a plane that was perpendicular to the B field vector. This would induce the maximum voltage across the lead (Eqn. 3). For each lead we scanned the probe along two different linear paths past the distal tip. One scan was along a *path *that was *parallel *to the "distal tip axis" (Fig. 3). This scan started with the probe tip 11 mm from the distal tip of the pacemaker and moves toward the tip, finishing 1 mm from the tip. We also scanned along a path that was *perpendicular *to the "distal tip axis" with the closest distance between probe tip and lead tip being 1 mm. The probe location terminology we used is based on the dimension between the nearest surface of the probe electrode tip and the nearest surface of the pacemaker lead tip. This method is non-invasive in terms of altering the leads or pulse generator under study.

Probe-tip orientation terminology is defined as follows: For each of these scans (perpendicular and parallel), we took data for two probe-tip orientations (Fig. 3). The *probe-tip orientations *are termed *normal *orientation and *aligned *orientation to define the relationship with the distal tip axis of the pacemaker lead. The distance between the closest point of the probe tip and the distal tip of the pacemaker lead under test was 1 mm when it passed directly by the lead tip in perpendicular and parallel orientations. The vector magnitude of the E-field at each point in the scan was calculated by computing the squared value of the magnitude of the voltage at each point, for each of the two probe tip orientations. Then the square root of the sum of the squares was computed to yield the vector magnitude of the E-field (Eqn. 4). Measurement of the third vector component (Z component) of the E-field (in a direction parallel to the B field vector) was not needed to determine the vector magnitude. This is because the value of the Z component of the E-field is zero in the plane that we placed the lead in.

### Measuring injected E-fields from pacemaker pulse generator voltages

We measured the E-field magnitude versus time at a fixed location, 1 mm from the distal tip of a lead with an active fixation tip while the proximal end was connected to an active pacemaker pulse generator (PG). This provided a good reference for E-field waveforms and amplitudes that are produced for clinically effective cardiac stimulation. The probe tips were aligned parallel to the distal tip axis of the lead. Two pacemaker pulse generators were used during separate measurements to deliver two different stimulation waveforms. The Medtronic KDR401 delivered 0.5 V pulses and the Guidant "Insignia" delivered 0.1 V pulses to a unipolar electrode configuration. We did not look at bipolar stimulation since it is known that this configuration produces less MRI magnetically-induced stimulation than unipolar stimulation [12]. For most measurements with pacemakers connected to a lead, the 1 kHz magnetic field from our gradient field simulator was present along with the pacemaker pulse. This allowed us to compare simultaneously the E-field injected by the pulse generator voltage with the E-field induced by the magnetic field. The two E-fields (magnetically induced and pulse-generator injected) could be partially separated by observing the probe voltage in the time domain with our oscilloscope. This method is non-invasive in terms of altering the leads or pulse generator under study. The magnetic field was on continuously, but the pulse generator was only on a fraction of the time, with a regular pulse rate. When the pulse generator was delivering a voltage, the E-field was a summation of the injected plus the magnetically induced fields. The injected pulse was much larger and readily observed. We also measured the E-field when only the pacemaker pulse was being delivered to the lead while no magnetic field was on. No unusual effects occurred for this case versus the case when both magnetic and injected E-fields occurred simultaneously.

### E-field near the distal tip versus E-field induced in saline - the E-ratio

For each lead (with no PG connected), we measured the vector magnitude of the E-field in saline with the probe 1 mm from the tip of the lead while the B field was on. We then measured the vector magnitude of the E-field at the same point with the lead removed from the saline ('empty' tank). We defined the E-ratio as the value of the magnetically-induced E-field at the tip of a lead divided by The E-field induced at this same location with no lead present. This E-ratio provides a measure of the enhancement of the E-field produced by a particular lead. The E-ratio data are shown in Table 1 for measurements with the large and the small probe. The absolute value of the E-field in the saline with no pacemaker lead present is easily calculated using equation 1. The E-field in the empty tank with saline, at the location where the tip of a lead would later be placed was 0.035 V/m. This information, along with the E-ratio allows us to calculate the absolute value of the E-field at the tip of a pacemaker lead.

**Table 1:** Values of the E-field measured near the distal tip of various leads with large probe (no pulse generator connected)

Lead	E-ratio (1 mm from distal tip) large probe	E-width (mm)	E-field at probe tip (V/m)
Simulated lead (insulated wire)	3.6	2.5	0.13
Active fixation tip	15.3	7.4	0.54
Tined Tip	18.7	3.2	0.65

Note: See the measurement uncertainty issues" section and the "Alteration of intended therapy by magnetically-induced E-fields" section.

## Results

We evaluated our data on E-field distributions induced by magnetic fields from our gradient field simulator. The E-field distribution was measured around the tips of each of the three real or simulated pacemaker leads. For one set of measurements, no pacemaker pulse generator was connected to the leads. The leads were configured in a loop submerged in saline. We measured along the two scan paths defined earlier; one that was parallel and one that was perpendicular to the distal tip axis. For each scan path the probe tips were oriented both normal to the distal tip axis and aligned to the distal tip axis. The vector magnitude (calculated from Eqn. 4) of the voltage from each of the two orientations was taken for each of the scans.

### Spatial distribution of E-field near the distal tip - falloff distance and width

**Figure 6:**
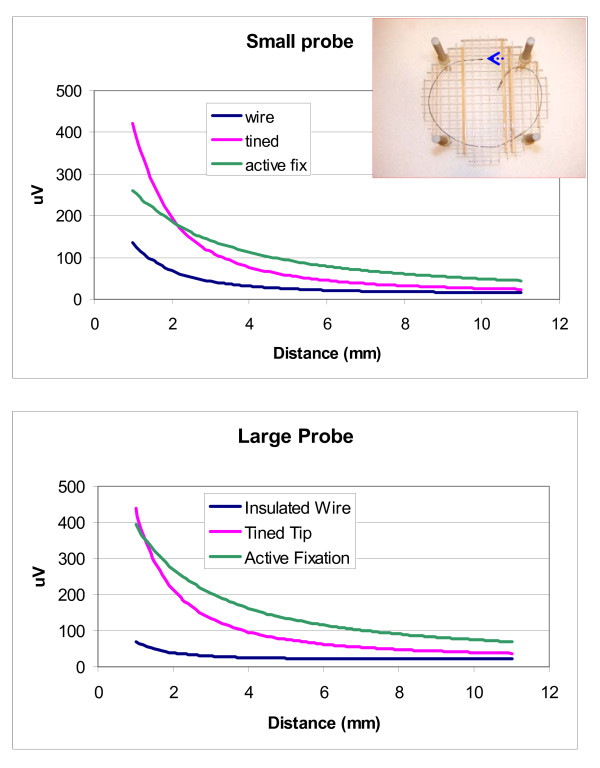
**Plots of raw data for the E-field measured while approaching tips of three leads**. The scan was parallel to the distal tip axis with the probe tips aligned with the distal tip axis.

**Figure 7:**
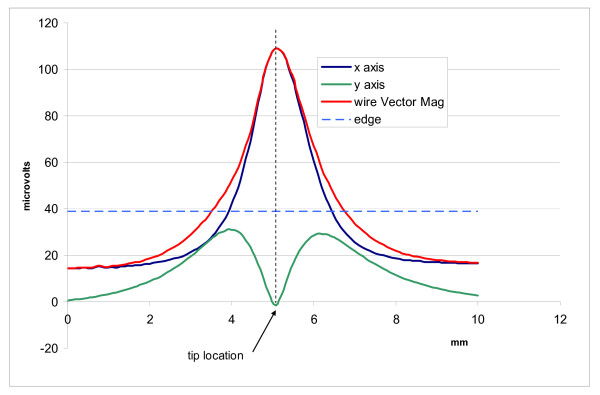
**Measured data for the simulated lead**. Vector magnitude of E- and 2 vector components measured with a scan perpendicular to the distal tip axis, passing 1 mm from the tip

**Figure 8:**
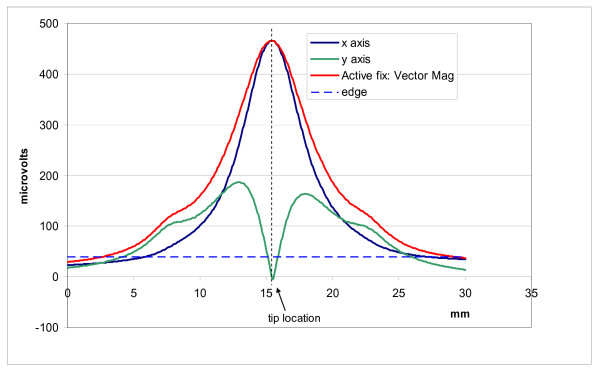
**Measured data for the active fixation lead**. Vector magnitude of E- and 2 vector components measured with a scan perpendicular to the distal tip axis, passing 1 mm from the tip

**Figure 9:**
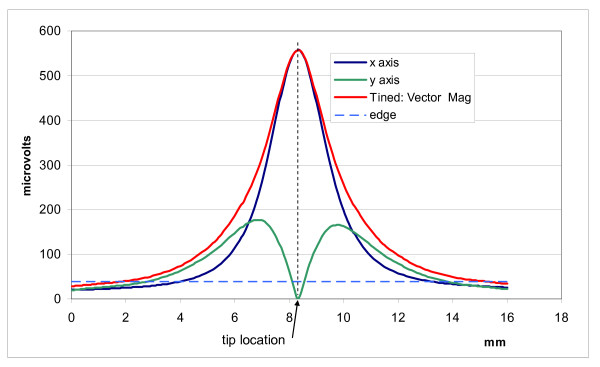
**Measured data for the tined tip lead**. Vector magnitude of E-field and 2 vector components measured with a scan perpendicular to the distal tip axis, passing 1 mm from the tip

We measured the E-field distribution near the tips of three leads by scanning our probe along two paths. One path was parallel and the other was perpendicular to the distal tip axis of each lead. Figure 6 displays raw data for each of our three leads for a scan parallel to the distal tip axis with the probe tips aligned with the distal tip axis. For measurement of the vector magnitude we only measured two components of the E-field. Figures 7, 8, and 9 show the vector magnitude (Eqn. 4) and two individual vector components for each lead, measured with a scan perpendicular to the distal tip axis using the large probe. The probe tip was scanned so it passed 1 mm from the lead tip at its closest point along the scan path. The line labeled 'edge' indicates the magnitude of the E-field at the edges (outer diameter) of the tank. This gives perspective on the enhancement of E at the lead tip relative to the maximum E in saline without the lead present. Data along this path provide the "width" of the E-field distribution. We defined the "width" (E-width) as the distance between two points along the scan where the value of the E- vector magnitude was half of the value at the measured maximum. Table 1 shows E-ratio, E-width and the absolute value of the E-field 1 mm from each of the three leads with the large probe. Table 2 compares the E-ratio for each lead as measured with the large probe versus the small probe.

**Table 2:** E-ratio for Large probe vs. Small probe

Lead	E-ratio Large probe	E-ratio Small probe	Small probe vs. Large probe
Simulated lead (insulated wire)	3.6	9.5	2.6
Active fixation tip	15.3	22.0	1.6
Tined Tip	18.7	30.4	1.6

Note: See the measurement uncertainty issues" section and the "Alteration of intended therapy by magnetically-induced E-fields" section.

### Injected E-field from Pulse generator

**Figure 10:**
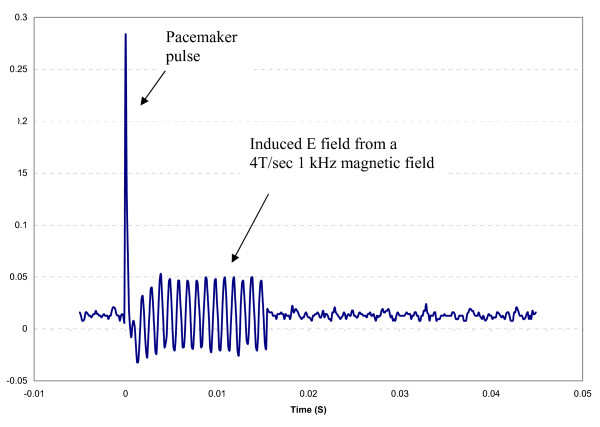
**Time domain plot of the E-field at the distal tip of a pacemaker lead connected to a pulse generator**.

Under normal operation an implantable pacemaker pulse generator (PG) delivers (injects) voltage pulses to the saline or surrounding medium at the distal tip of a lead connected to it. During exposures of an active pacemaker with a lead to a gradient field, two E- fields are produced near the lead tip. This occurs whether the lead is in a patient or in an in-vitro setup. These two E-fields are the injected plus the magnetically induced fields. We measured the E- field near the distal tip with the large probe for two cases, while each of two different pulse generators were connected during separate experiments to the proximal end of the active fixation lead, while a 1 kHz magnetic field was turned on. Two PGs were used in separate experiments, one delivering 0.1 V to the lead and the other delivering 0.5 V to the lead. The nearest wire of our 2-electrode E- field probe was 1.0 mm from the distal tip of the lead. The two electrodes of the probe were aligned with the distal tip axis. Figure 10 shows a time domain plot of the E-field at this location. When the implantable pacemaker's pulse generator was delivering a voltage to the lead, the E-field was a summation of the injected plus the magnetically induced fields. In addition, during a short time interval immediately after the PG output voltage pulse ended, a magnetically induced voltage (1 kHz sine wave) was still seen. This lasted for about 15 msec after the end of the pacemaker pulse. This is due to the implantable pacemaker PG circuitry providing a "recharge interval" which results in a low input impedance at the pulse generator implantable pacemaker connection to the proximal tip of the stimulating lead. No magnetically-induced voltage was seen at any other times during the PG output pulse cycle. This was documented previously [8,12]. During the implantable pacemaker pulse cycle, if a gradient field pulse is present, the E-field at the distal tip of a stimulating lead is the summation of the injected plus the magnetically induced fields.

**Figure 11:**
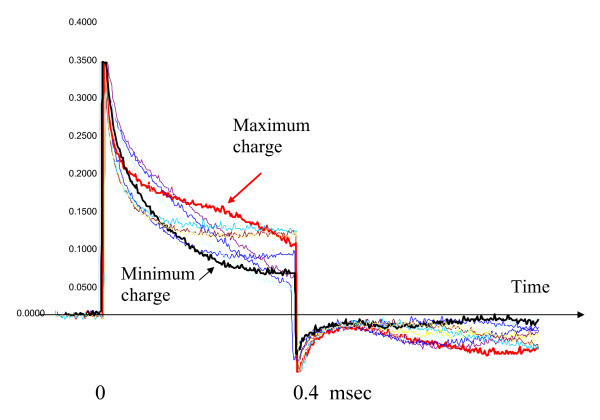
**E-field produced by combination of injected voltage from PG and a 4 T/sec magnetic field (1 kHz)**.

In a separate experiment we studied the deformation of the pacing pulse's waveform in saline by magnetically-induced E-field we used the maximum magnetic field we could generate to produce a 4 T/s exposure at 1 kHz. We monitored the intended stimulation waveform 1 mm from the distal tip with our probe. The waveform was significantly altered (decreased or increased) as shown in figure 11 during successive output pulses of the implantable pacemaker. This deformation will occur in clinical situations when the implantable pacemaker pulse coincides with the MR gradient pulse. The deformation can be much greater in clinical situations because a 30 - 100 T/S worst-case exposure is typical of clinical MR systems. We can linearly extrapolate our results from our 4 T/s exposure to any higher values. This involves taking the ratio of the clinical MRI system's slew rate versus our slew rate of 4 T/s. The exception to this is when voltages induced inside a pacemaker pulse generator circuitry are high enough to temporarily break down the semiconductor diodes and circuitry. Then magnetically induced E can occur at any time during the PG pulse cycle. This is discussed later in our paper.

## Discussion

### Measurements of magnetically induced E-fields in different leads and with different probe sizes

We measured the magnitude and spatial distribution of magnetically-induced E-fields near the tips of several leads. The active fixation lead and the tined tip lead each had a magnetically-induced E-field whose magnitude was quite similar at a location 1 mm from the tip. The E-field strengths for these leads was significantly higher than the E-field strength induced at the blunt cut tip of a simulated lead made of an insulated wire. The reason for this discrepancy is not known. This could be due to electrical properties of the wire, a smaller surface area, or other factors. The active fixation lead had the smallest tip diameter (less than 0.2 mm) and produced an E-field that was broader in distributed area (E-width) than the other leads. This lead had and E-width of 8.5 mm vs. 3.5 mm for the tined tip lead and 2.3 mm for the simulated lead. The E-field magnitude 1 mm from the two pacemaker leads did not change significantly when measured with the large (1.6 mm electrode spacing) or the small probe (0.9 mm electrode spacing). The simulated lead had a measured E-field that was significantly higher when measured 1 mm from the tip with the small probe. This change when measuring the E-field near the tip of the simulated lead must be due to steep spatial gradients in the E-field and the fact that the measured value is affected by spatial averaging or field perturbation as discussed below.

While we measured E-fields near pacemaker leads, the methods can be applied to leads of other stimulation devices such as neural stimulators (spinal cord implants and deep brain stimulators). This method is non-invasive in terms of altering the leads or pulse generator under study.

### Measurement uncertainty issues

We assessed measurement uncertainties of E-fields when the region of interest is extremely close to the tip of a simulated or actual pacemaker lead. Fields due to magnetic induction in a lead as well as fields due to voltage-injection from a pulse generator have large spatial gradients. For these cases the field being measured has significant changes in magnitude over a single millimeter (Fig. 6). There are several sources of uncertainties when measuring highly localized E-fields with a probe of finite dimensions. These include spatial averaging, field perturbation, probe positioning, and electromagnetic pickup.

Spatial averaging of the E-field occurs over the region occupied by our probe tips (1.6 mm or 0.9 mm for the large and small probes respectively). This averaging produces a reduction in the measured value of the E-field with large spatial variations relative to the true value of the E-field at an infinitesimal point (an underestimation). The uncertainty is greatest when the distance between the probe and the lead is on the same order as the probe tip size. Spatial averaging is not necessarily an undesirable effect. Clinically significant stimulation is affected by spatial averaging of applied E-fields. In electrically excitable tissues, stimulation is induced by the average E-field induced over some finite volume, not an infinitesimally small point. The measured "true" value of the E-field at an infinitesimal point can have uncertainties due to spatial averaging of the E-field by a probe with its two electrodes.

Another measurement uncertainty is due to perturbation of the E-field by metallic probe electrodes. This uncertainty is greatest when the distance between the probe and the lead is the same or less than the size of the total probe tip (both electrodes). This perturbation makes the measured value of the E-field lower than the true value of the E-field (an underestimation). This uncertainty diminishes with increasing separation between the probe and the pacemaker lead tip. The uncertainty from the combination of spatial averaging and field perturbation was estimated as being 50% at a separation distance that is equal to the outside dimensions of the total probe tip (1.6 mm or 0.9 mm for the large and small probes respectively). The combined uncertainties were determined by comparing the results of scans of the simulated lead with the small probe versus scans with the large probe. Also, we performed preliminary calculations of E with computational electromagnetics software. The results indicated that the probe's spatial averaging and field perturbation may produce measurement uncertainties that are greater than 50%. More validation of the computations must be performed before conclusions about worst case measurement uncertainties can be finalized.

A third measurement uncertainty occurs due to the error and repeatability for determining the true position of the probe tip with respect to a pacemaker lead. This can cause an underestimation or overestimation. For our mechanical system the positioning error and repeatability is about 0.1 mm. In addition, there is uncertainty due to a lack of repeatability in placing the probe tip at exactly the same location with respect to the lead tip being measured. The resulting uncertainty in the measured E-field value due to positioning variations is estimated as being 20% at a distance of 1 mm from the distal tip of the leads we evaluated. This uncertainty diminished as the separation increases. The estimates of the measurement uncertainties that are listed above are based on evaluation of spatial behavior of measured data, consideration of the size of our probe tips, and comparison of data from large and small probes. The 3 uncertainties above are common to measurements of both magnetically-induced E-fields and the pacemaker injected E-field.

There are additional uncertainties that are unique to the source of E-fields. For example, for magnetically-induced E-fields, electromagnetic pickup of the gradient magnetic field on wires at locations other than the probe tip introduces another uncertainty. Voltages are induced in the twisted pair of wires between the probe's distal tip and the preamplifier in the measurement system. This source of uncertainty should be identified and minimized. Measuring the E-field along a linear path extending from one edge of the tank to the other with the path passing through the center allows observation of this source of uncertainty. At the center of the saline tank, the measured voltage should be less than a few percent of the value of the voltage measured at the outer edges of each side of the tank. This voltage is measured with no implanted device lead in the tank, and with the two tips of the probe aligned with the E-field. Also, the value of the voltage measured at one side of the tank should be equal the voltage measured at the other side. This source of uncertainty can cause an underestimation or overestimation.

It should be noted that the issue of measuring E-fields with steep spatial variations is not unique to gradient-field MRI safety of implanted leads. The same issue has challenged those evaluating the RF-induced heating next to pacemaker lead tips by radiofrequency (RF) fields of MRI systems [7,13]. Significant errors in published measured values of maximum heating (due to E) are well known. This heating is caused by the magnetically induced E-fields from RF coils. These RF E-fields have very similar spatial distribution and fall off to insignificant values within a few millimeters of implanted leads.

Finally, for assessment of unintended stimulation by magnetic induction of E in leads, the effects of each of the uncertainties discussed above can be minimized or eliminated by determining the ratio of the simultaneous magnetically induced versus injected E-fields from an implantable pacemaker. This approach (comparison of magnetically induced E-fields versus injected E-fields at a fixed location near the distal tip) is discussed in the following section.

### Alteration of intended therapy by magnetically-induced E-fields

We compared the magnetically-induced E-fields at the distal tip of a lead with the "injected" E-field at the tip when the lead was connected to an active pulse generator of a cardiac pacemaker. We found that the intended pacemaker stimulation waveform (injected E-field) is degraded if a gradient pulse is coincident in time with the implantable pacemaker output pulse. We demonstrated the ability of relatively weak gradient fields (< 10 T/s) to alter therapy significantly. The individual gradient field pulses subtracted or added to the injected pulse, depending on their phase with respect to the injected pulse. The probability of this occurring in a clinical situation depends on the duration and time of occurrence of gradient pulses, relative to the occurrence of an implantable pacemaker pulse. E-fields were induced at the distal tip of a lead by gradient pulses only when the implantable pacemaker was delivering a voltage pulse or immediately after this pulse (within 15 ms). When no implantable pacemaker pulse was occurring, magnetically induced E-fields were not present near the lead tip. This is due to the fact that if both ends of the lead do not have a low-resistance contact with the saline, then virtually no magnetically-induced current can flow through the lead to the surrounding medium. When the proximal end of a lead is connected to a pacemaker pulse generator, a low-resistance path to the surrounding medium is provided by the electronic circuitry of the implantable pacemaker through the metallic case of the implantable pacemaker. A low resistance state in the circuitry occurs when a pulse is being generated or during an intentional low-impedance interval of about 10 to 20 ms following the pulse (depending on the implantable pacemaker design). When the low-impedance interval ends, the pulse generator circuitry is in a high resistance state and the E-field at the distal tip falls to very low levels. It is also possible for very intense gradient fields (e.g. 50 - 100 T/s) to produce a low-impedance state in the implantable pacemaker circuitry when the pacemaker is off. This can occur during non-destructive breakdown of various electronic components (e.g. diodes in the input protection circuit). This was demonstrated [12] using a clinical MRI system.

### Accurate assessment of the magnetically induced E-field

The comparison of gradient-field/magnetically induced E-fields versus pulse-generator injected E-fields at a fixed location near the distal tip provides a powerful method for assessing gradient induced stimulation with minimal measurement uncertainties. Simultaneous measurements of these E-fields allow the determination of the ratio of the magnitude of these two E-fields with high accuracy. The ratio eliminates errors due to measurement uncertainties caused by spatial averaging, perturbation of the E-field, and probe positioning. This elimination of errors occurs because measurements of each of the two E-fields have identical uncertainties, biased in the same direction.

### Conditions when potentially hazardous stimulation can be induced by a gradient field of an MRI system

Magnetically induced E-fields at the distal tip of a unipolar implantable pacemaker lead will usually occur only during or immediately after the pulse of the device's pulse generator. The intended stimulation waveform delivered by an implantable pacemaker pulse generator to a distal tip unipolar electrode can be significantly altered (decreased or increased). This is due to magnetically-induced E-field from a time-coincident MR gradient pulse combining with the injected pulse from the implantable pacemaker. Significant variations in the stimulating waveform shape were seen with only 4 T/s in experiments performed with our maximum available field (4 T/s). For an MRI system operating under normal conditions with a 30 to 100 T/s gradient field rate of change, significant changes in pacemaker stimulation voltages result when a gradient pulse is coincident (within about 30 ms) with a implantable pacemaker pulse. For a device other than an implantable pacemaker that uses long stimulating leads, similar conditions may exist. The magnitude of the magnetically induced E-field will depend on the impedance presented to the lead by the device's electronics, at the proximal end of the lead. In addition to the E-field amplitude, the duration and the time of occurrence of the gradient pulse from a clinical MRI system will determine the probability of unintended stimulation. The wide range of MRI sequences available to a clinician has significantly different duty cycles (percent of the time when a gradient pulse is generated). Some sequences such as echo planar imaging can produce continuous gradient pulses with a very high duty cycle (up to 100%) thus making the coincidence of a gradient pulse and a pacemaker pulse frequent. This could affect several pacemaker stimuli in a row, potentially canceling therapy delivery.

A simple way to reduce the possibility of unintended stimulation of a patient is to turn off their pacemaker during an MRI imaging session, provided they are not pacemaker dependent. This of course opens them to the risk of no available pacemaker therapy if they were to need it during the duration of the session.

### Future research needs for this work include several issues

Computational modeling of the experimental measurement system is highly desirable to compare with measured data. In the past, we have been limited in our ability to obtain reasonable results. The complexity of modeling a long wire (half a meter in length) and in large volume of saline becomes challenging when spatial resolution of a fraction of a millimeter is needed. This resolution is needed to model the tip and the conductive medium (saline) next to the tip. Here the E-field decays to insignificant levels a few millimeters from tip, so there are many cells in a square millimeter that need to be computed. Finally, the thin insulation layer requires a very large number of computational cells. Recent improvement in electromagnetics software and reduced costs should allow us to perform reasonably accurate modeling and present them in the near future.

A smaller probe with electrode tip spacing of less than 0.5 mm and fine wires is desirable to reduce measurement uncertainties from spatial averaging and field perturbation. A smaller probe produces a lower signal and thus reduces the signal to noise ratio of measurements. This limits detection of fields farther from the tip than a few millimeters.

## Conclusions

Potentially hazardous situations can exist due to gradient fields for certain situations. Unintended stimulation can be induced via abandoned leads if the proximal end is not "capped" with insulation by the cardiac surgeon. Unintended stimulation can also be induced in leads connected to a pulse generator with loss of hermetic seal at the connector block of the generator. For properly configured pacemakers the intended stimulation waveform delivered by a pacemaker pulse generator to a distal tip of unipolar electrode can be drastically altered (decreased or increased). This is of particular significance for certain high duty cycle gradient pulse sequences for pacemaker-dependent patients. This alteration is due to the magnetically-induced E-field from a time-coincident magnetic resonance (MR) gradient pulse. Findings of this work are applicable to other stimulation device with long implanted leads with small-area distal tips such as neurostimulators.

For bipolar leads, it is possible to recreate this effect for very high gradient fields (e.g. 100 T/s) due to non-destructive breakdown of diodes in the pulse generator. This effect was demonstrated recently [12] in a clinical MR system.

## Competing interests

The authors declare that they have no competing interests.

## Authors' contributions

HB conceived and designed experiments, analyzed data and drafted the manuscript and some figures. GM performed measurements, analyzed the data and created figures and data plots. All authors read and approved the final manuscript.
